# Opportunities and challenges for newborn screening and early diagnosis of rare diseases in Latin America

**DOI:** 10.3389/fgene.2022.1053559

**Published:** 2022-12-08

**Authors:** Roberto Giugliani, Silvia Castillo Taucher, Sylvia Hafez, Joao Bosco Oliveira, Mariana Rico-Restrepo, Paula Rozenfeld, Ignacio Zarante, Claudia Gonzaga-Jauregui

**Affiliations:** ^1^ Department of Genetics UFRGS, Medical Genetics Service HCPA, DASA and Casa dos Raros, Porto Alegre, Brazil; ^2^ Clinical Geneticist, Hospital Clínico Universidad de Chile, Clínica Alemana de Santiago, Santiago, Chile; ^3^ The NOA Project, Panama City, Panama; ^4^ Laboratório de Genética Molecular, Hospital Israelita Albert Einstein, Sao Paulo, Brazil; ^5^ Americas Health Foundation, Bogota, Colombia; ^6^ Instituto de Estudios Inmunológicos y Fisiopatológicos (IIFP), UNLP, CONICET, asociado CIC PBA, Facultad de Ciencias Exactas, Departamento de Ciencias Biológicas, La Plata, Argentina; ^7^ Instituto de Genética Humana, Pontificia Universidad Javeriana, Bogotá, Colombia; ^8^ International Laboratory for Human Genome Research, Laboratorio Internacional de Investigación sobre el Genoma Humano, Universidad Nacional Autónoma de México, Juriquilla, México

**Keywords:** newborn screening, early diagnosis, rare diseases, diagnostic odyssey, Latin America, genetics, genomics, molecular diagnosis

## Abstract

Rare diseases (RDs) cause considerable death and disability in Latin America. Still, there is no consensus on their definition across the region. Patients with RDs face a diagnostic odyssey to find a correct diagnosis, which may last many years and creates a burden for caregivers, healthcare systems, and society. These diagnostic delays have repercussions on the health and economic burden created by RDs and continue to represent an unmet medical need. This review analyzes barriers to the widespread adoption of newborn screening (NBS) programs and early diagnostic methods for RDs in Latin America and provides recommendations to achieve this critical objective. Increasing the adoption of NBS programs and promoting early diagnosis of RDs are the first steps to improving health outcomes for patients living with RDs. A coordinated, multistakeholder effort from leaders of patient organizations, government, industry, medical societies, academia, and healthcare services is required to increase the adoption of NBS programs. Patients’ best interests should remain the guiding principle for decisions regarding NBS implementation and early diagnosis for RDs.

## 1 Introduction

Latin America (LATAM) spreads over 20,000,000 km^2^ across 20 countries, plus 13 countries in the Caribbean region (Box 1), with approximately 620 million inhabitants overall (CELAC International, 2022). The region is vastly diverse culturally, geographically, politically, ethnically, and economically between and within countries, which is reflected in healthcare delivery and health-related indicators. Wealth distribution varies from countries with high Human Development Index (HDI), such as Chile, to low HDI, like Haiti (United Nations, 2020).

Box 1 **The 33 countries included in Latin America and the Caribbean by Region.**

**North America**: Mexico
**Central America**: Belize, Costa Rica, El Salvador, Guatemala, Honduras, Nicaragua, and Panama
**South America**: Argentina, Bolivia, Brazil, Colombia, Chile, Ecuador, Guyana, Paraguay, Peru, Suriname, Uruguay, and Venezuela
**Caribbean**: Antigua and Barbuda, Bahamas, Barbados, Cuba, Dominica, Granada, Haiti, Jamaica, Dominican Republican, San Cristobal and Nieves, San Vicente and the Granadinas, Santa Lucía, and Trinidad and Tobago

The definition of rare disease (RD) varies globally. In LATAM, there is no consensus on the definition of RDs. Some countries such as Bolivia, Ecuador, Paraguay, Peru, and Venezuela do not have a specific RD definition. Others, such as Brazil, consider RDs based on the World Health Organization (WHO) definition as affecting 65 or less per 100,000 individuals (Minister of Health, 2014). Argentina, Chile, Mexico, Panama, and Uruguay adhere to the European Union’s definition of <1 per 2,000 affected individuals (EURORDIS.Rare diseases, 2005), and Colombia defines RD as affecting <1 per 5,000 individuals. These diverse definitions also translate into the number of people living with a given RD varying across the region depending on whether their condition meets the different thresholds (Encina et al., 2019). The absence of a unified definition for the region creates challenges in estimating prevalence, creating standard policies and guidelines, integrating programs or registries, and allocating research funding.

An estimated 7,000–9,000 conditions are considered RDs, affecting around 6–8% of the world’s population (Ferreira, 2019). Although these disorders are individually infrequent in the general population, they affect roughly 300–350 million people worldwide (United Nations General Assembly, 2021). In LATAM, an estimated 35 million people live with a RD (Nguengang Wakap et al., 2020). If family members, caregivers, and health professionals are included, the number of people impacted by RDs is substantially higher. A genetic cause has been identified for over 6,000 of these disorders, providing the potential to determine a molecular genetic diagnosis. Therefore, it is possible to provide early detection, accurate diagnoses, and implement interventions to reduce the morbidity and mortality associated with these diseases (Gonzaga-Jauregui and Lupski, 2021).

Multiple efforts have been made for RDs from international entities, including a resolution in November 2021 by the United Nations calling for the implementation of national strategies to provide universal health coverage for patients with RDs, ensure access to diagnosis and treatment, increase research on RDs, and overcome inequality and exclusion gaps (United Nations General Assembly, 2021). However, despite advances in awareness of the medical and social issues surrounding RDs, inequality in the distribution of healthcare resources remains a reality for patients living with RDs in LATAM. Thus, the Americas Health Foundation (AHF) convened a panel of experts on RDs from Argentina, Brazil, Chile, Colombia, Mexico, and Panama for a multi-day conference to develop recommendations for increasing access to newborn screening programs (NBS) and early diagnosis of RDs in LATAM. This review aims to analyze the barriers to the widespread adoption of NBS programs and early diagnosis methods for RDs in LATAM and provide recommendations on achieving this critical objective.

## 2 Methods

AHF identified eight experts in RDs with backgrounds in newborn screening methods, genetics, and bioethics from Argentina, Brazil, Chile, Colombia, Mexico, and Panama. They were convened for a three-day virtual meeting on January 17-19 2022, to discuss the need for widespread access and adoption of newborn screening for RDs in LATAM. To select the panel, AHF conducted a literature review using PubMed, MEDLINE, and EMBASE to identify scientists and clinicians from the above countries who have had publications relating to RDs and molecular testing panels since 2016. Augmenting this search, AHF contacted LATAM opinion leaders from the medical field to corroborate the list of individuals who adequately represented the necessary fields of study. All the experts who attended the meeting are named authors of this paper. An AHF staff member moderated the discussion. The authors retain complete control over the content of the article.

AHF conducted a literature review using PubMed, MEDLINE, and EMBASE for any publications on newborn screening and molecular testing for RDs. The following search terms were used: “rare diseases,” “early diagnosis,” “newborn screening,” “Latin America,” “Mexico,” “Colombia,” “Argentina,” “Brazil,” “NBS,” “Panama,” and “molecular testing,” from 01/01/2016 to 04/10/2021. The identified articles were in English, Portuguese, and Spanish. Particular attention was paid to identifying literature and research in LATAM.

AHF developed specific questions to address the issues related to NBS for RDs in LATAM and assigned one to each panel member. A written response to each question was drafted by individual panel members based on literature review and personal expertise. The entire panel reviewed and edited each narrative during the three-day conference through numerous rounds of discussion until consensus was reached. For issues with disagreement among the panel, additional dialogues were held until all panel members agreed to the content included in this manuscript. The recommendations developed were based on the evidence collected, expert opinion, and professional experience and were approved by the entire panel. After the conference, the final manuscript was distributed by email to the panel for review and approval.

## 3 Results

### 3.1 Newborn screening and early diagnosis of rare diseases

RDs usually appear early in life, with approximately 70% having onset in the pediatric age, while an additional 12% can have onset in childhood or adulthood (Nguengang Wakap et al., 2020). Genetic RDs are the leading cause of death in children under 10 years of age (Harrison and Goodman, 2015). They are the leading cause of mortality and morbidity in neonatal and pediatric intensive care units in the United States, with a likely similar impact in LATAM (Harrison and Goodman, 2015).

RDs have the commonality of an extensive timeline to reach an accurate diagnosis, often referred to as a “diagnostic odyssey.” Although variable, the average delay between symptom onset and getting a diagnosis for many RDs is between 5 and 10 years (Vieira et al., 2008) during which time the health of these patients may deteriorate, and treatment opportunities may be missed. In countries where clinical genomic sequencing has been implemented, the diagnostic odyssey has been shortened by up to half for patients with suspected genetic disorders; however, with the traditional approach to diagnosis in most of LATAM, this delay has not decreased substantially despite increased awareness about RDs (Kuiper et al., 2018). An accurate and early diagnosis of RDs is essential for the patient, their family and healthcare systems. Even well-known RDs are diagnosed with unacceptable delays in many LATAM countries.

Obtaining a diagnosis is a determining factor for proper medical care, including treating symptoms, accessing therapies, and avoiding unnecessary interventions. On a personal level, a diagnosis allows patients with RDs and their families to make life-planning decisions, including on reproduction. A diagnosis also impacts a patient’s ability to be visible and recognized by public institutions. This may mean eligibility and access to social benefit programs, patient organizations (POs), and other support services, which may be diagnosis dependent. Health and social benefits include providing better care based on diagnostic-informed disease management, preventing comorbidities, facilitating access to social care and support, improving quality of life (QoL), and potentially increasing life span. Beyond individual outcomes, an early and accurate diagnosis remains imperative for epidemiology and planning for healthcare systems (Esquivel-Sada and Nguyen, 2018). Studies have demonstrated the utility of early and precise molecular diagnoses of newborns and children with RD to guide treatment and improve patient outcomes in the healthcare system (Pezzoli et al., 2021; Smedley et al., 2021).

NBS is a public health strategy conducted on newborns to identify potentially serious disorders before symptom onset or early enough to warrant a therapeutic intervention, reducing morbidity and mortality, and improving QoL. NBS is an essential vanguard for infant care and has provided vast improvements in the early diagnosis of many congenital diseases. In addition to advances in health outcomes, the severe clinical expression of the disease may be prevented and a reduction of healthcare expenditure with a benefit from a cost-effectiveness perspective may be achieved in some disorders (Cabello et al., 2021). However, varying limitations exist from country to country, which may range from continuity of care for the patient to cost and availability of these screening tests as budgets vary.

The importance of improving care from birth through the first week to decrease morbidity and mortality in children under 5 years was highlighted in the WHO’s recent objectives to ensure that every child in the world “survives and thrives to reach their full potential” [(World Health Organization, 2020), (Newborn Health, 2022)]. Congenital anomalies, either structural or functional, encompassing metabolic disorders, are a leading cause of neonatal death worldwide and contribute to chronic illness and disability in children. Along with comprehensive NBS programs, establishing or strengthening national programs for RD management is encouraged, emphasizing international reference networks, and the development of unified approaches for the prevention and care of congenital disorders (World Health Organization, 2010; Ferreira, 2019).

Currently, most NBS programs focus on the biochemical profiling of abnormal metabolites in newborn blood samples. The use of tandem mass spectrometry (TMS) has expanded biochemical panels, allowing the simultaneous screening of more than 50 inborn metabolic disorders (Friedman et al., 2017). Additionally, physical examinations and tests performed shortly after birth, such as pulse oximetry and hearing screenings, can identify congenital cardiac or hearing abnormalities in newborns that frequently have genetic underpinnings.

### 3.2 The landscape of newborn screening in Latin America

The implementation of NBS programs is generally delayed in low- and middle-income countries compared to higher-income nations due to economic, technical, and logistical constraints on top of each country’s social, cultural, and political background challenges. NBS originated in 1963 when Dr. Robert Guthrie created an assay to detect phenylketonuria (PKU). In the mid-1970s, the first NBS programs in LATAM began in Mexico and Brazil (Borrajo, 2021). More comprehensive NBS programs in the region were implemented in the mid-1980s, with Cuba launching a national NBS program in 1986. NBS pilot programs were started in other LATAM countries in the same decade. Later, national programs were implemented in Costa Rica (1990), Chile (1992) and Uruguay (1994) (Borrajo, 2007). Several other countries followed the same path and now NBS is a public health strategy covering a significant part of the newborns in the region, albeit with notable differences in terms of access, scope, and technologies (Queiruga et al., 2021).

**FIGURE 1:**
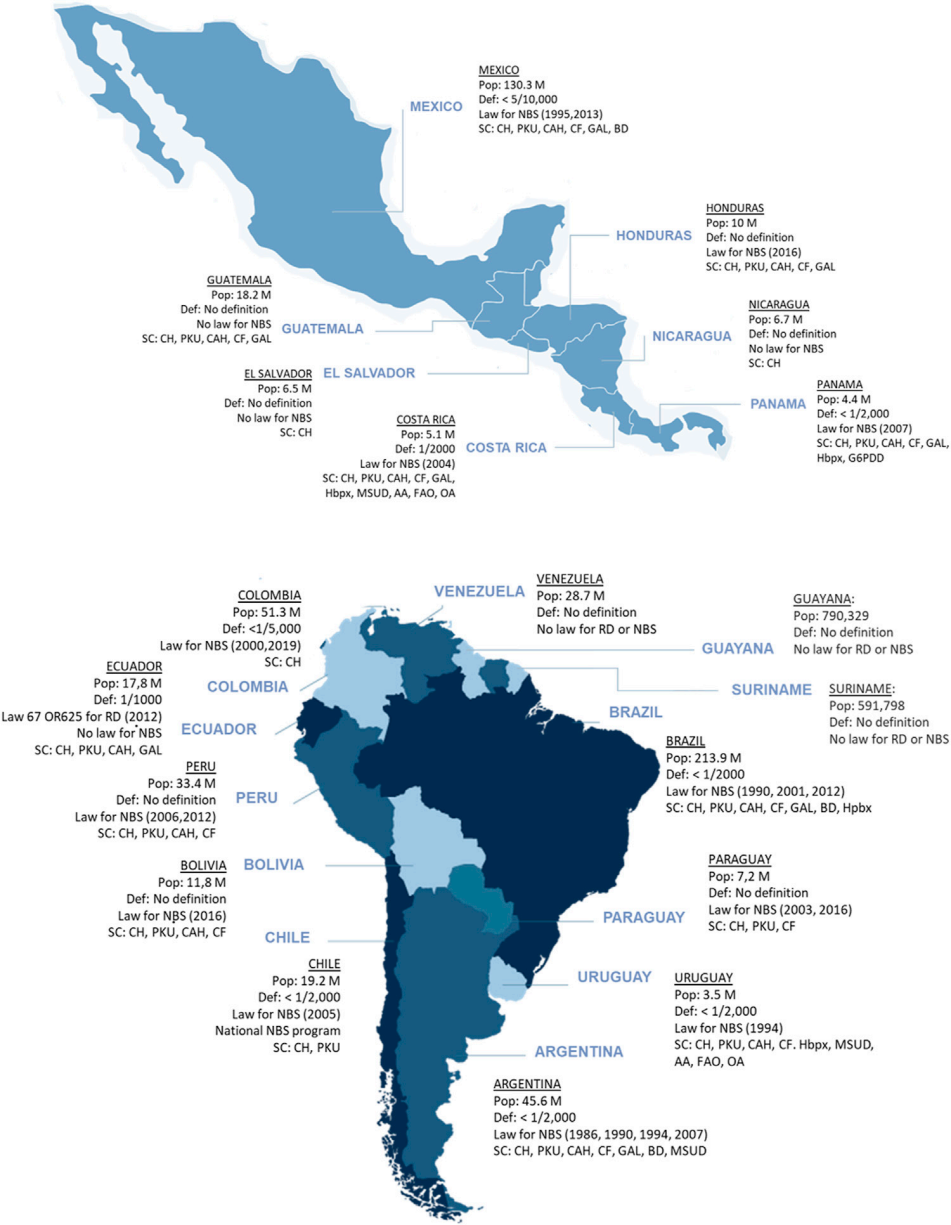
The landscape of RD definition and newborn screening in LATAM. Maps of Mexico, Central America and South America showing the 20 countries in the LATAM region and summarizing their population, whether they have a RD definition, whether NBS is mandated by national law and what year was that approved and the conditions for which the national NBS programs screen for. (Abbreviations: AA, other amino acid disorders; BIO, biotinidase deficiency; CAH, congenital adrenal hyperplasia; CF, cystic fibrosis; CH, congenital hypothyroidism; FAO, fatty acid oxidation disorders; G6PDD, glucose-6 phosphate dehydrogenase deficiency; GAL, galactosemia; Hbpx, hemoglobinopathies; MSUD, maple syrup urine disease; OA, organic acidurias; PKU, phenylketonuria).

Borrajo classified the NBS status of LATAM countries considering the following indicators: start dates, implementation modalities as organized programs, the panel of diseases screened, available testing technologies, coverage, legislation, and degree of development and success reached (Borrajo, 2007). According to these indicators, LATAM countries were classified into five groups, from fully established national programs to no programs. Sixteen countries have national or regional NBS programs (14 centrally coordinated and two conducted by regional healthcare providers). Thirteen countries have laws that establish mandatory NBS. Six countries provide 70–86% NBS coverage (Mexico, Colombia, Brazil, Panama, El Salvador, and Ecuador), and six more provide over 90% coverage (Cuba, Costa Rica, Chile, Uruguay, Argentina, and Paraguay). In general, the conditions most screened include CH (16 countries), PKU (14 countries), congenital adrenal hyperplasia (CAH), cystic fibrosis (CF) (12 countries each), and galactosemia (GAL) (8 countries). At a national level, assays for amino acids and acylcarnitines by TMS are implemented in only two countries, Costa Rica and Uruguay (Borrajo, 2007). Considering the countries with official NBS programs and their coverage percentage (Borrajo, 2007; Queiruga et al., 2021), it is possible to estimate that around 7.2 million newborns are screened per year in LATAM, representing approximately 72% of total births (US Census Bureau, 2021). An overview of major NBS programs in the world and LATAM is provided in Supplementary Table S1 and summarized in Figure 1. A description of NBS status in the countries represented by the authors of this review is provided below.

#### 3.2.1 Argentina

In Argentina, a national mandate for PKU screening was established in 1986. Currently, the federal NBS program looks for six conditions: CH, CF, GAL, biotinidase deficiency, and CAH. In Argentina, there are 20 regional NBS programs with varying coverage of disorders beyond government mandated ones (Governor of Colombia Office, 2022).

#### 3.2.2 Brazil

Extended NBS (ENBS) for other metabolic disorders through TMS screening has been implemented in a few states. Brazil started its nationwide NBS program in 2001 with reference centers that covered more than 80% of newborns. This program includes six main conditions: PKU, CH, hemoglobinopathies, CF, CAH, and biotinidase deficiency (Therrell et al., 2015). A law was passed in June 2021, mandating that all states implement ENBS by June 2022 and progressively expand the program to include lysosomal diseases, immunodeficiencies, and spinal muscular atrophy. However, existing challenges may hinder its implementation within the stipulated timeframe.

#### 3.2.3 Chile

The NBS program in Chile was approved in 1992 and was implemented stepwise in all 15 regions of the country by 1998. It currently covers CH and PKU. An ENBS pilot program is currently underway that aims to expand the number of conditions tested in Chile from 2 to 26 (Cabello et al., 2021).

#### 3.2.4 Colombia

Colombia established its national NBS program in 2000 to detect, confirm and treat CH in newborns (Peñaloza et al., 2020). By 2015, the program covered approximately 80% of all newborns in the country. In 2019, the legislation expanded the NBS program to include CH, PKU, CF, GAL, biotinidase deficiency, CAH, hemoglobinopathies, and visual, hearing, and cardiac screening (Presidencia, 2019). Additional disorders are being evaluated for inclusion into the ENBS program, including considerations for 33 disorders detected through TMS.

#### 3.2.5 Panama

Panama’s national NBS program was established in 2007 to detect PKU, CH, GAL, CAH, hemoglobinopathies, sickle cell disease, and glucose-6-phosphate dehydrogenase deficiency (No, 2009; Sánchez, 2021). The last two diseases were included due to their higher prevalence in Panama. In 2021, an amendment expanded the NBS program to include hearing, visual and cardiac screening, CF, and other unspecified inborn errors of metabolism (Secretary General of the National Assembly of Panama, 2021).

#### 3.2.6 Mexico

The Mexican healthcare system is incredibly complex and the lack of government regulations and guidelines for federally mandated NBS has resulted in a heterogeneous landscape of programs. Mexico was the first country in LATAM to implement an NBS program in 1974, which tested for PKU, CHT, and congenital toxoplasmosis. The toxoplasmosis testing was soon abandoned. In 1988, national legislation mandated NBS; however, despite evidence supporting the importance of screening for PKU, this disorder was also dropped, with only CH screening remaining (Vela-Amieva et al., 2009). In 2012, new guidelines emphasized the importance of ENBS covering at least CH, CAH, amino acid metabolism disorders, fatty acid metabolism disorders, GAL, hemoglobinopathies, severe combined immunodeficiency, and other disorders that represent a public health problem (Diario Oficial de la Federación, 2014). An ENBS program has been adopted in some institutions and by some states and efforts for national adoption of ENBS are ongoing. Currently, public and private institutions provide screening for additional conditions ranging from 4 to 70 at their discretion.

Despite disparities, the overall situation of NBS in LATAM indicates continuous improvement, especially in the last decade (Figure 1). Longstanding NBS programs (Chile, Costa Rica, Cuba, and Uruguay) cover over 99% of newborns. NBS programs in Brazil, Mexico, and Argentina have increased their screening panels but require education, follow-up, legislation, and management improvements. Ecuador, Peru, and Bolivia have shown important advances in recent years (Therrell et al., 2015).

### 3.3 Considerations for newborn screening implementation

#### 3.3.1 Stages of the newborn screening process

**FIGURE 2:**
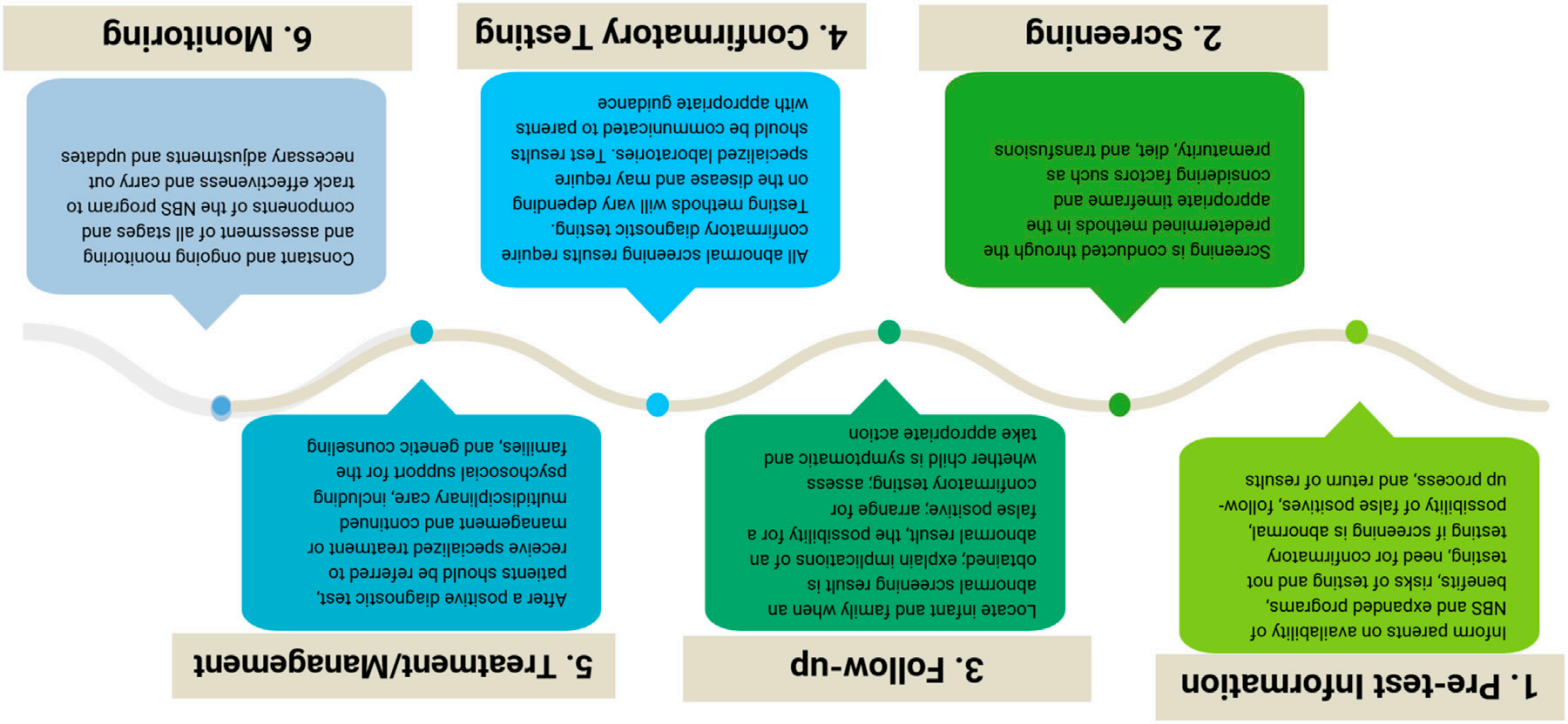
Stages of the newborn screening process. Schematic of the six stages and the corresponding considerations of the newborn screening (NBS) process.

NBS does not simply involve testing. NBS is a process that involves six stages and is generally organized and performed by public healthcare systems with the resources and authority to carry out universal screening (Therrell et al., 1992; Therrell, 2001). Throughout this process, many stakeholders are involved, including healthcare professionals, patients, families, and POs. Communication and interaction among stakeholders must occur before, during, and after the test. Each element of the screening process requires resources, sufficient training, standardized and accredited procedures, and quality controls that meet international standards. Figure 2 summarizes the stages of the NBS process.

The first stage is providing pretest information for parents. During this phase, parents must receive information about NBS, the possibility of expanded screening when available, and education on the benefits of early diagnosis for the diseases being screened, the risks for newborns who do not undergo testing, the need for confirmatory tests when screening is positive, the possibility of false positives, and the follow-up process and result delivery (Davis et al., 2006). This phase is not adequately carried out in most LATAM settings and must be improved.

The second stage is to conduct the testing through the determined and appropriate method, in the optimal timeframe. Pediatricians must be aware that certain factors can affect test results. These factors include incorrect age, prematurity, diet, and transfusions (Kaye et al., 2006).

The third stage involves following up on the results by locating newborns and their families when the results are positive or inconclusive. Although this may seem obvious, it often presents a challenge in LATAM, especially in rural settings where tracking patients and parents may be complex. Families must be informed of abnormal results as soon as possible so that confirmatory diagnostic tests can be performed and should receive guidance and support from a healthcare professional, usually a pediatrician or neonatologist, to explain the importance of positive screening results, the possibility of false positives and the need for confirmatory tests. At this stage, the treating physician must assess whether the child is in stable health and take appropriate action (Kemper et al., 2006).

The fourth stage is performing confirmatory diagnostic tests. Testing methods vary depending on the disease identified and often require specialized laboratories. A newborn who obtains an abnormal screening result will then undergo diagnostic testing and, if indicated, be referred for appropriate treatment and management.

The fifth stage is treatment and management. After a positive confirmatory test, patients are referred for specialized care. Genetic counseling must also be provided to the family to promote the detection of carrier status and inform the risk of recurrence and reproductive options. Continued multidisciplinary care is required for most patients with RDs.

The sixth stage is the constant and ongoing monitoring and assessment of all stages and components of the system: validation of the tests employed, measurement of the efficiency of the follow-up stage and interventions, and confirming the benefits for patients, their families, and society (Therrell et al., 2010; Hinton et al., 2016). At this stage, population coverage may be assessed. Treatment efficacy is determined, and problems with execution and maintenance are identified. The impact of diagnoses on families is also researched, as are the effects of screening on the population (EURORDIS.Rare diseases, 2005; Kemper et al., 2006).

#### 3.3.2 Rethinking criteria for newborn screening and early diagnosis of rare diseases

Historically, adding new disorders to NBS programs has been guided by the Wilson and Jungner principles proposed in 1968 (Wilson and Jungner, 1968). Broadly, these principles outline that to screen for a condition, the following criteria must be considered: 1) it must be an important health problem, 2) there should be an accepted treatment for identified patients, 3) facilities for diagnosis and treatment should be available, 4) it should have a recognizable early stage, 5) there should be a suitable test, 6) the test should be acceptable to the population, 7) its natural history should be adequately understood, 8) there should be an agreed policy on whom to treat as patients, 9) the cost of finding the patients, including the diagnosis and treatment, should be economically balanced with the expenditure on medical care, and 10) case-finding should be a continuous process. Considering these criteria, NBS programs would generally contemplate adding screening conditions only when the infrastructure, financial and human resources, and available treatments are in place to care for identified patients. However, technological advances such as TMS and, more recently, next-generation genomic sequencing (NGS) have dramatically increased the ability to detect and identify congenital disorders in newborns. Unfortunately, these technological advances are outpacing the healthcare systems’ ability to properly establish and provide the resources to manage and treat patients living with RDs. Nevertheless, the healthcare systems’ limitations and the lack of existing therapies for some RDs should not curtail the possibility of obtaining an early diagnosis for patients with RDs and their families. Although national healthcare systems in LATAM are unlikely to be adequately equipped to treat most RDs, obtaining an accurate diagnosis through NBS/ENBS or genomic sequencing approaches allows patients and their families to seek appropriate care and genetic counseling, enroll in clinical trials specific to their disease in their home country or abroad, obtain social benefits, and join disease support groups.

Leveraging experience and cost-effectiveness assessments from other programs and countries may help LATAM countries currently developing or updating their NBS/ENBS programs to effectively identify technologies and other disorders suitable for inclusion in their programs (Brower et al., 2022). To this end, it has been proposed to reevaluate and update the Wilson and Jungner principles to adapt to current testing strategies and possibilities, including adopting genome sequencing as part of the NBS process (Watson et al., 2006; Andermann et al., 2008; Andermann et al., 2011; Woerner et al., 2021). Some criteria to consider regarding the disorders to include in modern NBS programs are: 1) the condition’s incidence and prevalence, 2) the ability to detect the condition using the available technologies, 3) the sensitivity and specificity of the screening and diagnostic tests, 4) the disease burden, morbidity and mortality if left untreated, 5) the individual, familial, reproductive, and societal benefits of an early diagnosis and intervention. Additional factors such as treatment cost and availability, therapies to prevent adverse disease outcomes, and the cost and preparedness of the healthcare system to care for the patient may be considered but not be limiting factors.

NBS programs should identify opportunities to focus not only on treatable diseases, but also on medically actionable conditions. These include diseases where early interventions, which are not necessarily treatments or cures, lead to health gains for the patient, parents having reproductive options for future pregnancies, or avoiding a diagnostic odyssey. For untreatable but actionable conditions to be implemented in NBS/ENBS, the goal must expand from only benefiting the child clinically to helping the family. Such an expansion in scope will significantly increase the number and type of conditions eligible for screening.

### 3.4 The future of genomics in newborn screening and early diagnosis of rare diseases

The emergence of genomic sequencing technologies signaled a turning point in the understanding of RDs (Gonzaga-Jauregui et al., 2012; Gonzaga-Jauregui and Lupski, 2021). By harnessing these technologies, the possibility now exists to significantly reduce the time of the diagnostic odyssey that patients with rare genetic disorders endure, even if there is an unclear clinical diagnostic hypothesis. The adoption of NGS has improved the diagnostic rates of RDs over the past decade, used as a first-tier approach to achieve early diagnosis and as part of NBS (Gonzaga-Jauregui and Lupski, 2021). Nevertheless, much work remains to overcome current challenges in the implementation and interpretation of genomic variants in underrepresented populations, such as those of LATAM, to increase the diagnostic power of sequencing technologies. In addition to ensuring access, developing suitable infrastructure and databases linking genotypic and genomic information to clinical information is also essential to advance knowledge. Systematic implementation of NGS, and eventually exome sequencing or whole-genome sequencing in LATAM health systems can significantly improve the access of patients living with RDs to diagnosis.

The debate surrounding the use of NGS gene panels and exome or whole genome sequencing instead of or in addition to standard NBS methodologies has focused on the ability to accurately interpret genomic variants, costs for national healthcare systems, and ethical, legal, and social concerns (Yang et al., 2017; Downie et al., 2021). Pilot studies are looking at diagnostic rates and comparisons of false positive and false negative results between standard NBS and genomic sequencing approaches (Bodian et al., 2016; Wojcik et al., 2021; Kingsmore et al., 2022). While more detailed evaluation of the advantages and disadvantages of both methods is necessary, these initial studies suggest that genomic NBS for selected diseases would be valuable to complement ENBS programs. Together, these may provide the most comprehensive and accurate screening approach for newborn congenital disorders and rare genetic diseases.

### 3.5 Funding and policy considerations

Pilot programs are usually necessary to provide initial data for public policy development. These projects may be funded by private organizations, non-governmental organizations, or other funding sources. However, NBS programs require support from the nation’s Ministry of Health (MoH) to guarantee long-term viability. This may require participation as part of a national initiative, funded by and with full participation of government health authorities. There are other funding models in which patients may pay part or all the cost of ENBS out-of-pocket. Still, these models may impose an extra burden on disadvantaged populations and thereby increase health disparities (Padilla et al., 2009). Thus, program leaders must carefully develop appropriate costing data and financial planning from the outset. Industry-sponsored diagnosis programs may be helpful, and some are already in place to provide access to confirmatory testing after a positive NBS result or if a RD is suspected. These programs are also used to identify patients for clinical trials and/or provide treatment options and market research for RD-approved drugs. However, to be sustainable at the national level, NBS and early diagnostic strategies must successfully intersect with public healthcare (Therrell and Padilla, 2014). Partnerships within the RD ecosystem among POs, research centers, pharmaceutical companies, and governments at the local and international levels are crucial.

### 3.6 The role of patient organizations

PO and advocacy groups aim to bridge the gap between government, industry, healthcare, and patients. As ambassadors for different RDs, they understand and have lived through the importance of an early diagnosis and how life-changing it can be for patients and their families. In addition to policy changes and infrastructure, LATAM countries require efforts dedicated to education and advocacy for which the PO’s role becomes imperative. As prominent actors in the RD ecosystem, POs advocate for their respective diseases before healthcare organizations and government entities.

POs can increase awareness of RDs by educating policymakers and leaders of healthcare institutions on the importance of early diagnosis and the associated challenges of disease progression. They can also provide feedback to policy leaders to empower them to create or tailor legislation to the RD community’s needs. Additionally, the medical and academic communities can benefit significantly from the input and feedback these groups provide on the needs and journey of a patient living with a RD and their family.

POs can also create awareness within the medical community about ultra-RDs, increasing physicians’ likelihood of suspecting and diagnosing such diseases. Although some RDs are relatively frequent, for others, patients may be one of the only cases in a country or region.

POs can educate and empower parents to request NBS from their healthcare providers and advocate for ENBS and diagnosis programs from health authorities. In fact, the first NBS programs in the US that screened for PKU were the result of advocacy activities by families and parents of children with intellectual disabilities (Therrell, 2001). Advocacy can be achieved by educating the general population about their rights, available options, and the benefits of NBS and early diagnosis programs. The efforts of POs have elicited changes to increase RD awareness and advocate for novel therapies and improved policies. Likewise, these efforts will be crucial to achieving the full implementation and potential of NBS and early diagnosis programs for RDs in LATAM.

### 3.7 Challenges and barriers to early diagnosis of rare diseases and widespread adoption of newborn screening in Latin America

The primary health challenges in each of the LATAM countries vary from fighting malnutrition and providing basic needs, such as clean water, to implementing ENBS. (Borrajo, 2021). Thus, any plans to implement widespread NBS or early diagnosis programs for RDs in the region must consider the vast disparities and contrasting priorities of the member countries. The successful planning and implementation of a national NBS/ENBS program require many components, and stakeholders should consider and implement each of these parts, adjusted to their local reality (Therrell and Padilla, 2018; Padilla et al., 2020). One of the main obstacles to NBS and early diagnosis of RDs in the region is the patient’s navigation through the fragmented healthcare systems. In most countries, there is a lack of integrated healthcare delivery models that support the diagnosis, treatment, and management of RDs, with few or no comprehensive care centers for RDs. Furthermore, patients sometimes get lost in the NBS process because they are born outside of a healthcare institution, postpartum discharge occurs 24–48 h after birth, and remote sampling programs are not widely available. If a screening test produces an abnormal result, patients may get lost for social, geographical, or logistic reasons. A list of challenges with recommendations suggested by this panel can be found in Table 1.

**TABLE 1:** Challenges and recommendations for newborn screening implementation in Latin America.

Challenges	Recommendations
1) Some countries do not have designated legislation establishing NBS programs and early diagnosis strategies	**Policy Changes and Development**
	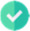 Five countries in LATAM without RD legislation must create a national policy guaranteeing and regulating the care of patients with RDs
	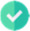 Countries that already have dedicated RD legislation and policies must ensure NBS and early diagnosis of RDs are prioritized and continuously expanded within the legislature. Any screening is better than no screening, but at a minimum, CH and PKU must be conducted with the maximum coverage possible
	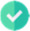 Create a MoH advisory committee that includes decision-makers from the government, payers, health institutions, public and private laboratories, academia, bioethicists, medical and scientific associations, and POs to develop national screening plans and advise the government on policy development based on the RD community’s needs
	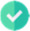 Carry out a consensus among LATAM experts in RDs to adopt a unified definition for RDs to facilitate collaboration within the region. This effort must include perspectives from medical societies, academia, and POs. Follow-up efforts must then be organized to advocate for the adoption of this definition by all countries in the region
2) Insufficient education and awareness among stakeholders including policymakers, parents, and the general public on the importance of NBS programs and early diagnosis strategies	**Advocacy and Awareness**
	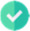 Awareness campaigns targeting all stakeholders, including policymakers, the public, private and public entities, and the healthcare community should advocate for:
	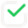 The need to develop, expand, improve, and provide equal access to NBS and early diagnosis for RDs as well as to its life-changing impact for patients and their families
	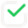 The importance of equal opportunities, social justice, and protection, and eliminating discrimination and stigma for people living with RDs and their families to alleviate some of the challenges they face
	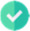 Expecting parents must be educated and informed of the benefits and limitations of NBS, their right to access it, and options for expanded screening
	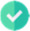 Parents or caretakers of children with RDs must be educated on care, management, and the nature of the disease. Psychosocial support must be provided in the face of a diagnosis
3) Lack of training of the healthcare community (primary care professionals, midwives, gynecologists, pediatricians, neonatologists, among others) in fundamental knowledge about genetics, RDs and early diagnosis for RDs, including NBS/ENBS, and its impact on health indicators	**Training for the healthcare community**
	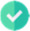 All healthcare professionals with neonatal contact (including gynecologists, nurses, midwives, genetic counselors, neonatologists, pediatricians, clinical geneticists, and primary care physicians) should receive adequate training on:
4) Shortage or absence of specialists in the RD field, including nurses, genetic counselors, medical geneticists, and pediatric subspecialties. This shortage is especially exacerbated in terms of genetic counselors and medical geneticists because in most LATAM countries, only physicians can provide genetic counselling, there are few training programs for this specialty, and few job opportunities in the region	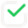 RDs according to their level of care. Specifically, because they are the first point of contact with neonates, every pediatrician and neonatologist must receive training on when to suspect a RD and appropriate referral situations to get an early diagnosis and prompt treatment
	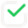 Handling communication with families before, during, and after NBS or RD diagnosis, considering the benefits of testing, the uncertainties and limitations surrounding NBS and other diagnostic methods, and the implications of a diagnosis
	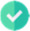 Address the shortage of specialists in the RD field, including specialized nurses, genetic counselors, medical geneticists, and pediatric subspecialists by developing training programs in these fields with corresponding job opportunities and incentives
	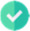 Broadening regulations on which professionals can train to provide genetic counselling could increase the number of these specialists

## 4 Conclusion

Keeping up with technological advances to screen, diagnose, treat, and manage RDs is a global challenge. Early identification of patients with RDs through NBS provides a population-wide benefit and advantage to prevent disability, morbidity, and early mortality. Increasing the adoption of NBS/ENBS programs and promoting early diagnosis of RDs are the first steps to improving health outcomes for patients living with RDs. Children’s best interests should remain the guiding principle for the basis of decisions regarding NBS implementation and early diagnosis for RDs. (Borrajo, 2021). A coordinated, multistakeholder effort from leaders of POs, government, industry, medical societies, academia, and healthcare services is required to increase the adoption of NBS/ENBS and early diagnosis programs. These strategies must strive to provide patients and their families with a diagnosis to seek appropriate care or support within their country or abroad. Developing programs in LATAM must continuously leverage the progress made in other regions. Through shared efforts, NBS and early diagnosis approaches for RDs will continue to improve health and QoL for patients and their families. Due to the vast heterogeneity in LATAM, recommendations must be tailored and adapted according to each country’s and healthcare system’s capacities. Although the recommendations presented here were developed to address the contemporary challenges in LATAM, they may be applied and extrapolated to other resource-limited regions and settings.
