# Supplementary material for: Opportunities and challenges for newborn screening and early diagnosis of rare diseases in Latin America
Source: Front Genet. 2022 Dec 8;13:1053559. doi: 10.3389/fgene.2022.1053559 (PMC9773081; doi:10.3389/fgene.2022.1053559)
Supplement: Supplementary file 1 [file DataSheet1.PDF]

Supplementary Table 1. Representative newborn screening programs in the world and Latin America.

Contry/ Region	Year first implemented	Current number of screened conditions	Public/private access	Observations
Europe	1960s (most recent in 2000)	Number of screened conditions variable among countries, from 1 in Montenegro to 29 in Austria	Public national by country	High variability among countries in access and number of disorders that are included in NBS programs
USA	1960s	Recommended Uniform Screening Panel (RUSP) for 61 disorders but variable among states ranging from 28 to 73 screened disorders.	Public by individual state	
Canada	1960s	Only 3 conditions screened across the country (CH, PKU, and MCADD). Additional conditions screened vary per province/territory.	Public by individual province or territory	
Mexico	1974	Only CH is required nationwide. Screening for 4 to 70 disorders in addition to CH variable depending on hospital setting. Other conditions include PKU, CAH, CF, GAL, BTD, and G6PDD)	Public and private options	Variable number of disorders assayed depending on institution. ENBS starting to be applied in some states screening for 67 disorders
Argentina	1986	Screening for 6 disorders (CH, PKU, CAH, CF, GAL, BTD)	Public nationwide mandated	Some cities screen for additional disorders according to local laws

Contry/ Region	Year first implemented	Current number of screened conditions	Public/private access	Observations
Bolivia	2006	4 disorders (CH, PKU, CAH, and CF)	Public	Varies per region
Brazil	2007	Currently screens for 6 conditions (CH, PKU, CAH, CF, BTD, and Haemoglobinopathies)	Public nationwide	Addition of more disorders to national NBS being explored, while ENBS for metabolic disorders available in some states.
Chile	1992	Currently screens for 2 conditions (CH and PKU)	Public	ENBS pilot undergoing to expand to 26 conditions
Colombia	2000	Only CH is screened for nationwide. PKU, CF, GAL, BTD, CAH and Haemoglobinopathies added in 2019 as part of the basic NBS program.	Public nationwide mandated; private options available.	ENBS pilot program being evaluated to screen for 33 total disorders. Additional conditions being screened by private health insurance providers.
Costa Rica	1990	Screening for 29 conditions including CH, PKU, CAH, CF, GAL, Haemoglobinopathies, MSUD, other amino acid disorders, fatty acid oxidation disorders, and organic acidurias	Public nationwide mandated	Among the most comprehensive public programs in LATAM and with greater coverage.
Ecuador	2011	4 disorders (CH, PKU, CAH, GAL)	Public nationwide	
Honduras	2016	5 disorders (CH, PKU, CAH, GAL, CF)	Public	Variable coverage

Contry/ Region	Year first implemented	Current number of screened conditions	Public/private access	Observations
Panama	2007	8 disorders (CH, PKU, CAH, GAL, CF, Haemoglobinopathies, Sickle cell disease, G6PDD)	Public nationwide mandated	Expansion of NBS program evaluated in 2021. High prevalence of G6PDD in population.
Paraguay	2004	3 disorders (CH, PKU, CF)	Public nationwide	
Peru	2012	5 disorders (CH, PKU, GAL, CAH, CF) and hearing loss	Public nationwide	
Uruguay	1994	28 disorders including CH, PKU, CAH, CF, Haemoglobinopathies, and additional 23 metabolic conditions including MSUD, other amino acid disorders, fatty acid oxidation disorders, and organic acidurias	Public nationwide	Among the most comprehensive public programs in LATAM and with greater coverage.
Venezuela	1999	2 disorders included (CH and PKU)	Public nationwide	Additional conditions being evaluated for addition to national NBS program. Limited screening through tandem MS approaches.

Abbreviations: NBS: newborn screening; ENBS: expanded newborn screening; CH: congenital hypothyroidism; PKU: phenylketonuria; MCADD: medium chain acyl-CoA dehydrogenase deficiency; CF: cystic fibrosis; GAL: galactosemia; CAH: congenital adrenal hyperplasia; BTD: biotinidase deficiency; MSUD: maple-syrup urine disease; G6PDD:
